# Development of an In-House ELISA for Serological Detection of Equine Herpesvirus-1/4 Antibodies in Turkish Horses

**DOI:** 10.3390/ani15172523

**Published:** 2025-08-27

**Authors:** İlker Şahinkesen, Seval Bilge-Dağalp

**Affiliations:** 1 Diagen Biotechnological Systems Health Services and Automation Industry, Ankara 06070, Türkiye; 2 Department of Virology, Faculty of Veterinary Medicine, Ankara University, Ankara 06070, Türkiye

**Keywords:** antibody, EHV-1, EHV-4, ELISA, equine serology

## Abstract

Equine herpesvirus 1 (EHV-1) and Equine herpesvirus 4 (EHV-4) are major viral pathogens of horses that contribute to respiratory disease, abortion, and neurological disorders, leading to economic losses in the equine industry. Accurate serological diagnosis is critical for disease surveillance and control. This study aimed to develop and validate an in-house enzyme-linked immunosorbent assay (ELISA) for detecting antibodies against EHV-1 and EHV-4 in horse sera. Serum samples previously confirmed by virus neutralization test (VNT) and a commercial ELISA were used to optimize the assay. Cut-off values were established using negative controls, and 155 serum samples were tested. The developed ELISA demonstrated 80.64% positivity for EHV-1 and 79.35% for EHV-4. When compared to VNT, the assay showed 85% specificity and 100% sensitivity. A high similarity (99%) was also observed when compared with the commercial ELISA. The overall seroprevalence was found to be 54.19% for EHV-1 and 75.48% for EHV-4, with an SN_50_ value of 1/10 for both. The validation results confirmed high repeatability and reliability across plates. These findings suggest that the developed in-house ELISA is a practical, cost-effective, and accurate tool for the serological monitoring of EHV-1 and EHV-4 infections in horse populations.

## Simple Summary

Equine herpesviruses are significant infectious agents that can cause respiratory illness, miscarriages, and nervous system problems in horses, leading to serious economic losses. Early detection of these viruses is essential to control their spread and protect animal health. In this study, we developed and evaluated a laboratory method that detects specific antibodies in horse blood to identify previous exposure to the viruses. The method was compared with standard reference tests to ensure accuracy and reliability. In addition to validating the method, this study also provided valuable information about the current situation of these infections among horses in Türkiye. This approach offers a cost-effective and dependable tool for monitoring herd immunity and supporting veterinarians and researchers in disease control efforts.

## 1. Introduction

Herpesvirus infections in horses pose significant economic and epidemiological challenges, threatening both individual animal health and the global equine industry [1]. To date, nine different herpesviruses have been identified in equids. Among these, EHV-1 (Equine abortion virus), EHV-3 (Equine coital exanthema virus), EHV-4 (Equine rhinopneumonitis virus), EHV-6 (Asinine herpesvirus 1), EHV-8 (Asinine herpesvirus 3), and EHV-9 (Gazelle herpesvirus 1) are classified within the Varicellovirus genus of the Alphaherpesvirinae subfamily, while EHV-2, EHV-5, and EHV-7 (Asinine herpesvirus 2) belong to the *Gammaherpesvirinae* subfamily [2,3]. In terms of host range, horses serve as the natural hosts for EHV-1, -2, -3, -4, and -5; donkeys for EHV-6, -7, and -8; and gazelles for EHV-9 [4,5]. Among these viruses, Equine herpesvirus type 1 (EHV-1) and Equine herpesvirus type 4 (EHV-4) are the most prevalent in horses and are responsible for the most severe clinical outcomes. Clinically, EHV-1 is associated with abortion storms, neonatal deaths, respiratory disease, and neurological manifestations such as equine herpesvirus myeloencephalopathy (EHM). EHV-4 primarily causes respiratory tract infections, though it is occasionally isolated from abortion cases [6,7]. These viruses typically enter the host through the respiratory tract, causing primary infection characterized by nasal discharge, fever, conjunctivitis, anorexia, and depression [8,9,10]. Following the initial infection, and similar to other herpesviruses, both EHV-1 and EHV-4 establish lifelong latency in the host. Stress factors such as transportation, pregnancy, or corticosteroid administration can trigger reactivation of the latent virus [11,12,13]. Reactivation is reported to be more frequent with EHV-4 than EHV-1, possibly due to the seasonal nature of EHV-1 infections. Once reactivated, the virus is shed and can spread rapidly among susceptible animals, potentially leading to outbreaks [5].

The diameter of mature virions ranges from approximately 120 to 300 nm, depending on the presence of the envelope and tegument layers. The viral genome is enclosed within an icosahedral capsid measuring about 100–110 nm in diameter and composed of 162 capsomers. The tegument fills the space between the envelope and nucleocapsid, and includes proteins and enzymes required to initiate viral replication [14,15]. The viral glycoproteins embedded in the envelope are critical for mediating host cell attachment, membrane fusion, intracellular spread, and host tropism [16]. These glycoproteins are also the primary targets of the host immune response, as their location allows direct interaction with immune system components [5]. A total of 12 glycoproteins have been identified in the envelope of EHV-1 and EHV-4. Among them, gB, gD, gH, gK, and gL are essential for viral replication, while gC, gE, gI, gG, gM, gN, and gp300 are considered non-essential but play roles in immune evasion, viral dissemination, and pathogenesis [14].

For the serological diagnosis of these viruses, complement fixation (CF), virus neutralization (VN), and ELISA have been described in paired, acute, and convalescent sera samples taken from suspected animals. A four-fold or greater rise in virus-specific antibody titer within paired sera has been proven to indicate these infections [1]. The protective titer against these infections has been reported as 1/64 [17]. In another study, it was reported that 4 to 8-fold more neutralizing antibodies were produced against EHV-4 than EHV-1 in animals vaccinated with a single dose of the EHV-4 vaccine. It has been suggested that an antibody titer of 1/256 and above, detected in horse sera, may indicate acute EHV-1/4 infection [18]. Both viruses are antigenically similar and serologically cannot be distinguished from each other using conventional methods as CFT and VNT [19,20]. For this reason, a type-specific ELISA for EHV-1/4 epitopes located at the C-terminus of glycoprotein G (gG) has been developed [21].

The presence of EHV-1/4 infection in Türkiye has been demonstrated by many studies, and EHV-1/4 vaccination is mandatory for horses, especially in public enterprises and boarding stud farms. Several seroepidemiological studies conducted in Türkiye have reported varying levels of EHV-1 and EHV-4 seropositivity in horses, donkeys, and mules across different provinces. Reported EHV-1 prevalence ranged from 3.7% to 52.3%, while EHV-4 prevalence ranged from 56.9% to 81.7% [22,23,24,25]. These findings indicate that the detection of antibodies in equids can serve as an indicator of infection or, in vaccinated animals, a vaccine-related response. Particularly for rapid diagnosis, the ELISA technique is more advantageous than the neutralization test.

The aim of this study was to develop a broadly applicable and cost-effective ELISA capable of determining the overall seroprevalence of EHV-1 and EHV-4 infections in equids, including both infected and vaccinated animals. To enhance the clinical relevance and diagnostic accuracy of the assay under local conditions, a field-derived EHV-1 strain was employed as the coating antigen, allowing for a more accurate reflection of circulating viral strains among equine populations in Türkiye. Additionally, a reference strain of EHV-4 was utilized to investigate potential cross-reactivity, an issue frequently mentioned but insufficiently quantified in previous studies. By addressing this gap, the present study not only proposes a practical diagnostic tool for epidemiological and immunization monitoring but also contributes valuable data to the existing literature regarding serological cross-reactivity between EHV-1 and EHV-4.

## 2. Materials and Methods

### 2.1. Animal Samples

In this study, 200 horse sera samples were collected from horses for the optimization steps and statistical evaluation of the in-house ELISA. Of these, 105 sera samples were collected with ethical committee permission (2022-7-6). The remaining 95 sera samples were kindly provided by Prof. Dr. Veysel Soydal Ataseven and Prof. Dr. Yakup Yıldırım.

The purpose of collecting serum samples was to provide negative and positive controls for the optimization stages of the in-house ELISA. Additionally, the study aimed to evaluate and validate the in-house ELISA and determine the seroprevalence of EHV-1/4 antibody status among Turkish horses. Precolostral foal sera and horse sera that confirmed to be negative by virus neutralization test (VNT), commercial ELISA served as the negative control sera. Positive control sera consisted of sera samples that were positive for EHV-1/4 in both the VN assay and commercial ELISA.

### 2.2. Viruses

In this research, EHV-1/4 provided by the Veterinary Control Central Research Institute Laboratory Türkiye, Ankara were used in the VN assay and the development of the in-house ELISA. EHV-1 was a field isolate, and EHV-4 was a reference strain (ED0440). Both viruses were grown in the equine dermis (ED) cell line (ATCC, Manassas, VA, USA), aliquoted into centrifuge tubes, and stored in a −80 °C deep freeze.

### 2.3. Virus Neutralization Assay

Whole sera samples were tested in the VN assay with both viruses according to the method described by Frey and Liess (1971) [22]. Briefly, the inactivated sera samples were diluted (1/2) and then transferred to 2 wells of 96-well cell culture plates (Greiner Bio-One, Kremsmünster, Austria) in a volume of 50 μL. An equal volume of virus diluted to 100 TCID_50_ was added. For the virus control wells, 50 μL of Dulbecco’s Modified Eagle Medium, low glucose (DMEM-LG: Sartorius, Göttingen, Germany) and 50 μL of 100 TCID_50_ virus dilution were added to the wells. For cell control wells, 100 μL of medium with fetal calf serum (FCS; Gibco, Thermo Fisher Scientific, Waltham, MA, USA) was added. The plates were incubated for 1 h at 37 °C in an incubator with 5% CO_2_. After incubation, a cell suspension prepared at 300,000 cells/mL was added to all wells. The VN assay was evaluated based on cytopathological changes observed under a tissue culture microscope at the end of the third day for EHV-1 and the fifth day for EHV-4.

To determine the antibody titer in sera samples, an SN_50_ assay was performed to assess the presence of antibodies against EHV-1/4. Briefly, the sera samples were diluted twofold (1/4, 1/8,1/16,1/32,1/64), and then the VN assay was conducted according to the method reported by Frey and Liess (1971) [22].

### 2.4. Development of In-House ELISA

#### 2.4.1. Purification of the Viruses

For this purpose, high-titer virus preparations were treated with 15%, 9%, 7%, and 5% (*v*/*v*) PEG6000 supplemented with 3.75 M NaCl, and the mixtures were incubated at 4 °C for 16–18 h. Subsequently, ultracentrifugation (CP100NX, Hitachi, Tokyo, Japan) was performed at 50,000 rpm and 4 °C for two hours to remove PEG residues. The precipitated virus pellet was resuspended with PBS and kept in the refrigerator (4 °C) overnight. It was then stored in a −20 °C deep freezer until use [23].

In the second method, the virus, produced and stored at high titer, was directly subjected to ultracentrifugation. After the virus was transferred to ultracentrifuge tubes with the help of a syringe, it was centrifuged at 50,000 rpm and 4 °C for four hours. The tube was then cut with a scalpel, the supernatant was discarded, and the virus pellet formed at the bottom was resuspended in PBS and allowed to dissolve at 4 °C overnight [24].

#### 2.4.2. Inactivation of the Viruses

For this purpose, purified viruses were inactivated using thermal heat blocks at 56 °C for 30 min. To confirm inactivation, virus inoculation into cell cultures was carried out for three passages. When it was confirmed that there was no growth at the end of the passages, measurement of protein amounts was started [25].

#### 2.4.3. Detection of Antigen Integrity Post-Inactivation

To evaluate the structural integrity of the inactivated viral antigens used in the ELISA, two complementary analyses were performed: sodium dodecyl sulfate polyacrylamide gel electrophoresis (SDS-PAGE) and dynamic light scattering (DLS).

For SDS-PAGE, purified and inactivated EHV-1 and EHV-4 virions were mixed with Laemmli sample buffer, boiled for 5 min, and loaded onto 12% polyacrylamide gels under denaturing conditions. Electrophoresis was carried out at 120 V for approximately 90 min. Following separation, the gels were stained using Coomassie Brilliant Blue R-250 to visualize viral protein bands. This technique was used to assess the preservation of major structural proteins, including envelope glycoproteins and capsid components [26].

DLS was performed using a Zetasizer Pro (Malvern Panalytical, Malvern, Worcestershire, UK) to determine the particle size distribution and homogeneity of the inactivated virus preparations. For analysis, 1 mL of each antigen suspension was transferred into DTS0012 disposable cuvettes (Malvern Panalytical, Malvern, Worcestershire, UK) and equilibrated at 25 °C prior to measurement. Samples were measured in triplicate. The average hydrodynamic diameter and polydispersity index (PDI) were recorded. DLS analysis was employed to verify whether the physical integrity and dispersity of the virions were preserved following the inactivation process.

#### 2.4.4. Viral Protein Quantification

The amount of viral proteins was calculated using the Bradford assay (Serva Electrophoresis GmbH, Heidelberg, Germany) [27].

#### 2.4.5. Optimization of the In-House ELISA

For this purpose, checkerboard titrations were performed twice for each virus, as described by Crowther (2008) [28]. The first checkerboard titration aimed to find the appropriate concentration of antigen and antibody. The second titration aimed to determine the correct antibody and conjugate concentration while keeping the antigen concentration constant.

For antigen and antibody titration; a twofold dilution of the antigen was made, starting with 20 µg/mL and ending with 0.0097 µg/mL from the first to the twelfth well of the plate. The positive antibody for EHV-1/4 (*n* = 10) titrations were performed, starting with a 1/50 dilution to 1/6400 dilution, from wells A to H of the plate.

For positive antibody for EHV-1/4 and conjugate titration; twofold dilution of the positive antibody were made, starting with 1/50 and ending at 1/256,000 from the first to the tenth well of the plate.

The conjugate “Goat anti-Horse IgG (T) (Bio-Rad Laboratories, Hercules, CA, USA) can be diluted from 1/10,000 to 1/100,000 for ELISA, according to the provider’s description. In our assay, it was diluated slightly out of range, from 1/5000 to 1/125,000.

#### 2.4.6. The Determination of the Blocking Solution and Concentration

Optimization was made with different concentrations of skimmed milk (Serva Electrophoresis GmbH, Heidelberg, Germany) and BSA (Sigma-Aldrich, St. Louis, MO, USA) for blocking. These agents were added to the plate in a volume of 200 μL after the antigen coating was completed. Blocking was performed by keeping the plates on a shaker for two hours at room temperature. To optimize, BSA was used in concentrations of 2%, 1%, 0.5%, and 0.25%, and skimmed milk was used in concentrations of 2%, 5%, 7%, and 10%, respectively [29].

#### 2.4.7. Validation of the In-House ELISA

For this purpose, the cut-off value was first calculated using the formula: “mean of negative controls + standard deviation of 3× negative controls × 1.1.” In this study, negative controls were obtained from precolostral serum samples of foals (*n* = 15) and horse sera confirmed to be negative by virus neutralization test (VNT) and a commercial ELISA (*n* = 10). After determining the cut-off value, the sera samples above it were considered positive, and sera samples below it were considered negative. After evaluating all sera samples with the in-house ELISA, diagnostic specificity and sensitivity were calculated by comparison with the VN test, which is the gold standard in diagnosis.

After calculating diagnostic specificity and sensitivity, the coefficient of validation was determined following the WOAH guidelines (2023) [30]. This step aimed to assess the repeatability of the test. Two plates (SPL Life Sciences, Gaithersburg, MD, USA) coated at the optimal density of antigen were used separately for EHV-1 and EHV-4 and tested with positive sera samples. The coefficient of validation (CV%) was then calculated using the formula: “Standard deviation/Mean × 100.”

#### 2.4.8. Testing Sera Samples with Commercial ELISA

In order to compare the results obtained from horse sera tested with VNT/SN_50_ tests and the developed in-house ELISA kit, and to determine their similarities, a commercial ELISA kit (Svanova, Uppsala, Sweden; Cat. No. SV-104901) was used to detect antibodies against the proteins encoded by the EHV-1/4 gG gene region. The test was performed following the procedure specified by the manufacturer.

## 3. Results

### 3.1. Virus Inoculation and Virus Titration Assay

**Figure 1:**
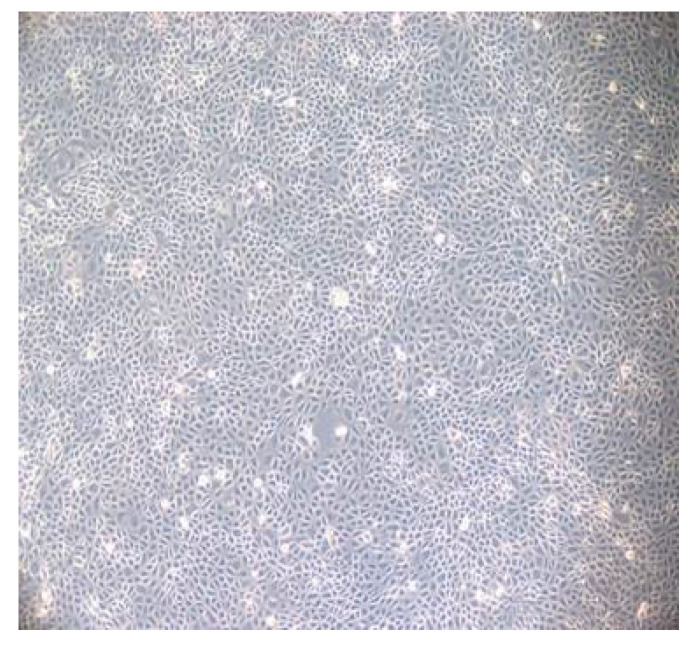
Morphology of a non-infected cell monolayer of the equine dermis cell line.

**Figure 2:**
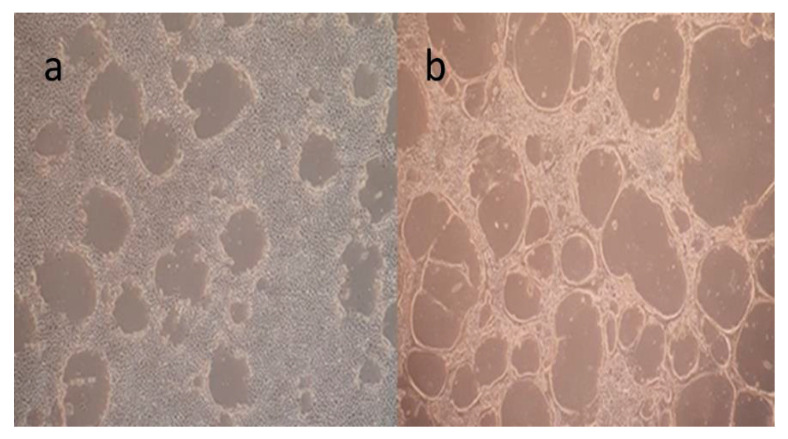
(**a**) EHV-1 growth 18 h after inoculation; (**b**) EHV-1 growth 24 h after inoculation.

**Figure 3:**
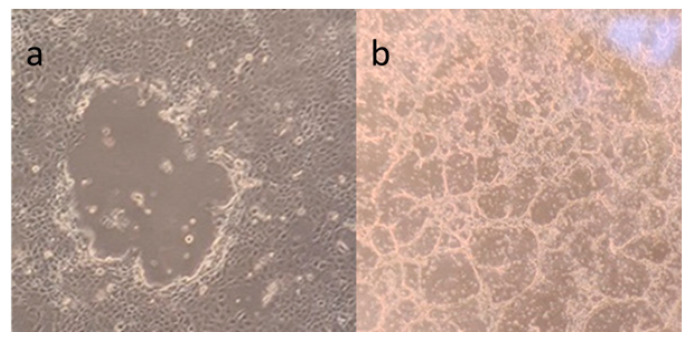
(**a**) EHV-4 growth 48 h after inoculation; (**b**) EHV-4 growth 120 h after inoculation.

The equine dermis (ED) cell line was cultivated for virus cultivation and viral neutralization (VN) assays (Figure 1). EHV-1 was inoculated and grown in the ED cell line and when it reached the third passage, its titer was calculated and stored in a −80 °C deep freezer. (Figure 2a,b) EHV-4 was also inoculated and grown in the ED cell line, and when it reached the eight passage, its titer was calculated and stored in a −80 °C deep freezer. (Figure 3a,b) The titer of EHV-1/4 was calculated as 10^5.25^ TCID_50_ per 0.1 mL, according to Frey and Liess’s (1971) method [22].

### 3.2. VN and SN_50_ Assay

The VN assay was performed separately for both viruses on all sera samples. Of the 155 sera samples tested, 93 (60%) were positive for EHV-1 antibodies, and 120 (77.41%) were positive for EHV-4 antibodies. Additionally, 45 sera samples (22.5%) could not be evaluated due to toxic effects, contamination, or other factors. As a result of SN_50_ tests performed on positive sera (*n* = 120 for EHV-4, *n* = 93 for EHV-1), the average antibody titer for both viruses was determined to be 1/10. Among vaccinated animals (*n* = 98), the average titer was 1/8, whereas naturally infected animals exhibited an average titer of 1/12. The overall seroprevalence of the sampled animals was determined as 54.19% (84/155) for EHV-1 with an SN_50_ value of 1/10, and 75.48% (117/150) for EHV-4 with an SN_50_ value of 1/10.

### 3.3. Assessment of Antigen Integrity Post-Inactivation

**Figure 4:**
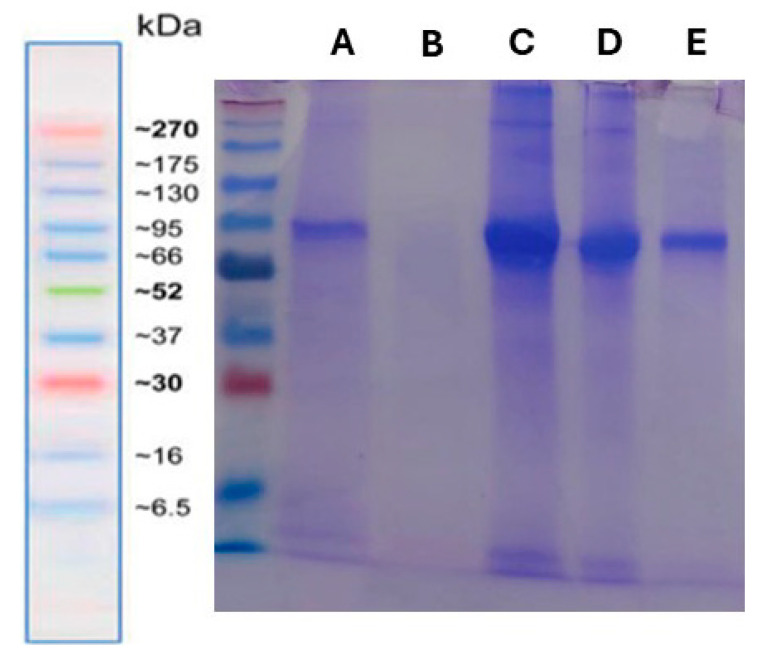
SDS-PAGE analysis of inactivated EHV-1 and EHV-4 virions under denaturing conditions. Viral antigen preparations were loaded as follows: Lane A—EHV-1 (0.1 mg/mL), Lane B—Negative control (buffer only), Lane C—EHV-1 (0.5 mg/mL), Lane D—EHV-4 (0.5 mg/mL), Lane E—EHV-4 (0.1 mg/mL). Prominent protein bands were observed at approximately 62 kDa and 95 kDa, corresponding to putative glycoproteins such as gC and gB, respectively. Molecular weight markers (left) are indicated in kDa.

The protein profiles of inactivated EHV-1 and EHV-4 virions were evaluated by SDS-PAGE under denaturing conditions. Distinct bands were observed at approximately 175 kDa and 270 kDa, likely corresponding to highly glycosylated forms of gB and possibly the major capsid protein (MCP). A prominent and dense band appeared at around 62 kDa, which may represent a combination of envelope glycoproteins such as gC and gE, or other tegument-associated proteins (Figure 4). These findings suggest that the overall structural protein integrity of the virions was largely preserved following the inactivation process.

DLS analysis was conducted in triplicate to evaluate the particle size distribution and homogeneity of inactivated EHV-1 and EHV-4 preparations. The Z-average hydrodynamic diameter was measured as 126 nm for EHV-1 (PDI: 0.24) and 130 nm for EHV-4 (PDI: 0.30). Both viral preparations exhibited similar size profiles, indicating that the inactivation process did not significantly alter the overall physical integrity or dispersity of the virions.

### 3.4. Evaluation of In-House ELISA

First, viral protein purification was performed on the viruses cultivated in large volumes, and two methods were evaluated for this purpose. With the PEG method, the viral protein concentration was quite low (0.312 μg/mL) in the Bradford assay, so this method was abondened. In the direct ultracentrifugation method, the viral protein concentration was detected at 2 mg/mL for EHV-1 and 1.5 mg/mL for EHV-4. These amounts were found sufficient for further experimentation.

#### 3.4.1. Optimization of In-House ELISA

**Figure 5:**
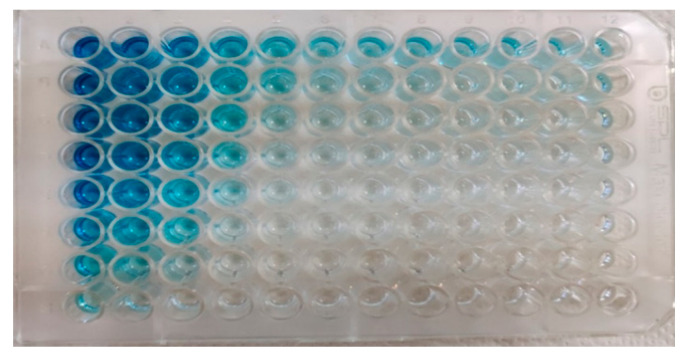
Antigen–antibody checkerboard titration for EHV-1.

As mentioned earlier, checkerboard titration experiments were conducted to determine the optimal concentrations of the antigen (purified EHV-1 or EHV-4 virus separately), antibody (from EHV-1/4 positive sera), and conjugate (Biorad, Hercules, CA, USA, AAI38P). The optimal concentration of antigen was found to be 2.5 μg/mL for EHV-1, (Figure 5) and 1.25 μg/mL for EHV-4. The optimal antibody concentration was determined to be a 1/100 sera dilution for EHV-1 and a 1/200 sera dilution for EHV-4. The optimal conjugate dilution was found to be 1/75,000 for both viruses.

After optimizing the in-house ELISA, the cut-off value was determined using negative sera samples from precolostral foals. The cut-off value was found to be 0.205 OD for EHV-1, and 0.174 OD for EHV-4.

Regarding the potential effects of lot-to-lot variation and long-term antigen storage on assay performance, all virus stocks were prepared at a standardized concentration (1 mL at 1 mg/mL) and stored at −80 °C in 50% glycerol to maintain antigen stability throughout the study. Before each assay, the antigens were coated onto ELISA plates at their predetermined optimal concentrations. To ensure consistency between batches, a quality control ELISA was conducted using a positive reference serum. Only antigen lots yielding OD values within a ±5% deviation from the target range were accepted for use. This approach was implemented to ensure both the reproducibility and reliability of the assay over time.

#### 3.4.2. Evaluation of Sera Samples with In-House ELISA, Comparison with VN Assay and Commercial ELISA

For this purpose, 155 sera samples were selected for testing with the commercial ELISA due to the high number of toxic sera samples (22.59%). Thus, the specificity and sensitivity of our in-house ELISA were determined using these 155 sera samples.

In the EHV-1 in-house ELISA, 80.64% (125/155) of the samples tested positive, whereas in the EHV-4 in-house ELISA, (123/155) 79.35% tested positive.

In the VN assay, 60% (93/155) of the samples were positive for EHV-1, as shown in (Table 1), and 77.41% (120/155) were positive for EHV-4, as shown in (Table 2).

In the commercial ELISA, 72.90% (113/155) of sera samples were found positive for EHV-1, while 12.25% (19/155) of sera samples were found doubtful. For EHV-4, 85.80% (133/155) of sera samples were found positive.

Due to serological cross-reactivity between EHV-1 and EHV-4 viruses, the whole virus-based in-house ELISA and VN assay could not distinguish antibody responses for either EHV-1 or EHV-4. Thus, as shown in (Table 3), the antibody responses in the VN assay and in-house ELISA recognized EHV-1 and EHV-4 together without distinction. The specificity and sensitivity were determined based on this situation with the VN assay serving as the gold standard for these calculations. Consequently, the specificity of in-house ELISA was determined to be 85%, and the sensitivity was determined to be 100%. The similarity rate with the commercial ELISA was determined to be 99%.

In this study, sera samples were collected from both vaccinated and unvaccinated horses. There were 98 vaccinated and 57 unvaccinated sera samples that could be evaluated using the in-house ELISA, VN assay, and commercial ELISA. The results are shown in (Table 4).

The overall seroprevalence of the sampled animals, determined using EHV-1 VNT, EHV-1 commercial ELISA, and EHV-1 in-house ELISA, was 54.19% (84/155) for EHV-1 with an SN_50_ value of 1/10 and 75.48% for EHV-4 with an SN_50_ value of 1/10. (Supplementary Materials). Although the same serum samples was used across both SN and ELISA-based assays, no significant correlation was observed between SN_50_ titers and OD values obtained from the ELISA tests, suggesting that these assays may detect different antibody populations or reflect different stages of the immune response.

#### 3.4.3. Validation of In-House ELISA

To assess the repeatability of the developed in-house ELISA, the coefficient of variation (C.V.%) was first determined. The coefficient of validation indicates the consistency of the results when variables such as antigen, antibody, and conjugate are kept constant during the development of a serological assay. The lower C.V. percentage, the more consistent the results are. The highest accepted coefficient of validation for a serological assay has been reported to be 15% [31]. Two ELISA plates were used for each virus, and the protocol was performed as described by WOAH (2023) [30]. As a result, the C.V. of the first plate for EHV-1 was 4.19%, and the C.V. of the second plate was 3.75%. The C.V. of the first plate for EHV-4 was calculated as 3.01%, and the C.V. of the second plate was calculated as 2.87%. The fact that these C.V. values are lower than the acceptance value of 15% shows that the developed ELISA is well-validated.

The second step in the validation process is to determine the diagnostic specificity and sensitivity, which were previously determined as 85% specificity and 100% sensitivity.

## 4. Discussion

To diagnose EHV-1 and EHV-4, two conventional methods, known as the CF assay and the VN assay, are commonly used. These methods cannot differentiate between two viruses due to serological cross-reactions. Performing these methods requires live viruses, suitable cells, and a full-fledged laboratory, and they are time-consuming. For example, the VN assay requires three days for EHV-1 and five days for EHV-4. The other serological method for diagnosis of EHV-1/4 is ELISA. This technique offers several advantages over VN assays: it is simpler, faster, and can evaluate a large number of samples within 2 to 3 h. In addition, certain commercial ELISA kits can distinguish between the viruses. However, there are a limited number of commercial ELISA kits available for detecting EHV-1/4 antibodies recently. In this commercial ELISA, only a small number of samples (*n* = 52) can be evaluated.

In this study, an in-house ELISA was developed to detect the presence of antibodies against both agents in animals either vaccinated with the inactivated vaccine containing both agents, or unvaccinated and naturally infected. For this purpose, the viruses were cultivated in large volumes and then purified to use as antigens for coating ELISA. Therefore, as previously mentioned, two methods were evaluated. In the PEG purification method, the viral load was detected to be very low (0.312 μg/mL). This was attributed to the possibility that PEG particulates might damage the viral receptors. Due to this reason, direct ultracentrifugation has been used for purification. With this method, a sufficient amount of viral protein (2 mg/mL) was obtained for further research.

The structural integrity and purification efficiency of the viral preparations were evaluated using SDS-PAGE and DLS analyses. SDS-PAGE revealed distinct bands corresponding to viral glycoproteins and capsid proteins, indicating that the inactivation process did not compromise the structural integrity of the virions. DLS measurements showed Z-average hydrodynamic diameters of 126 nm for EHV-1 and 130 nm for EHV-4, with PDI values of 0.24 and 0.30, respectively. These findings indicate a moderately uniform particle distribution and suggest the potential applicability of the purified viral antigens in downstream ELISA development.

In ELISA, carbonate-bicarbonate buffer and PBS are used as coating solutions. The choice of solution should be made based on a pH that is one to two degrees above the isoelectric point of the antigen [28]. The pH of PBS is 7.4, while the pH of the carbonate-bicarbonate buffer is 9.2, respectively. The isoelectric point of herpesviruses has been detected as pH 5.0 in a study [32]. Therefore, PBS was chosen for the coating solution. In this study, BSA and skimmed milk were evaluated as blocking agents at different concentrations. The optimal reagent and concentration were found to be 1% BSA and 5% skimmed milk. For further studies, skimmed milk was selected because a study by Konishi et al. (2010) showed that human antibodies bind the BSA, resulting in false positives when developing indirect ELISA for the Japanese encephalitis virus [33].

Checkerboard titration tests were conducted to determine the optimal concentrations of antigen, antibody, and conjugate, as previously mentioned. The results showed that when the conjugate was diluted more intensely to 1/10,000 instead of the optimal dilution of 1/75,000, even a sera dilution of 1/256,000 tested positive. Similarly, when the antigen was diluted more intensely to 20 μg/mL instead of the optimal concentration of 2.5 μg/mL for EHV-1, even a sera dilution of 1/6400 yielded a positive result. These results prove the importance of a checkerboard titration test for the development of ELISA.

To determine the presence or absence of antibodies against EHV-1 and EHV-4 in horse sera and to compare the developed ELISA with the VN assay, both methods were performed. For this purpose, 200 horse sera samples were provided. However, 45 sera samples (22.5%) could not be evaluated using the VN assay due to toxic effects from the horse sera and/or contamination. This result indicates that ELISA has advantages over the VN assay, even though the VN assay is considered the gold standard, as it allows for the evaluation of these samples in ELISA. Additionally, the serological cross-reaction between EHV-1 and EHV-4 was encountered more frequently in the in-house ELISA, which contains the whole virus, since ELISA is more sensitive than the VN assay.

During the EHV-4 outbreak in Germany, [17] showed that a great number of the foal sera positive by commercial ELISA could not be detected as positive by the VN assay. They attributed this discrepancy to a delayed neutralizing antibody response and/or the maternal antibodies preventing the formation of neutralizing antibodies [17]. This finding also proved that the VN assay more disadvantages than ELISA. In a study conducted in Morocco, [34] compared the seroepidemiology of EHV-1/4 infections using the VN assay and ELISA. Samples were collected from a total of 405 horses, 163 of which were unvaccinated and 242 of which were vaccinated with the EHV-1 inactive vaccine [34]. The commercial ELISA detected antibodies against EHV-4 in 100% of the horses; for EHV-1, 21.8% of vaccinated animals and 12.8% of unvaccinated animals tested positive. In the VN assay, all animals evaluated for EHV-4 were found positive with an average antibody titer of 1/45. For EHV-1, 90.5% of ELISA-positive samples and 53.6% of ELISA-negative samples tested positive in the VN assay. Researchers attributed these results to antigenic cross-reaction between the two viruses. The high seroprevalence observed for EHV-4 was attributed to the endemic circulation of the virus [34]. Similarly, EHV-4 infections can occur throughout the year, while EHV-1 infections occur during the winter months, which is the gestation period for horses [5]. Our study revealed that the seroprevalence of EHV-4 was higher than that of EHV-1, with overall rates of 54.19% (84/155) for EHV-1 and 75.48% for EHV-4 among the sampled animals. Although serological cross-reactions between EHV-1 and EHV-4 were observed, the in-house ELISA effectively detected antibody responses against both viruses together, as intended.

Several studies have aimed to develop ELISA using whole EHV-1/4 as antigens. Carvalho et al. (2000) developed an in-house ELISA using complete EHV-1 as an antigen and reported 94.7% sensitivity and 100% specificity when comparing the results with the VN assay [35]. In another study, an in-house ELISA was developed using whole EHV-4 as an antigen [36]. These studies indicated that the EHV-1 and EHV-4 cannot be distinguished when using the whole virus as an antigen due to serological cross-reaction between the viruses; however, both can be detected together.

Due to serological cross-reaction between EHV-1 and EHV-4, both the whole virus-based in-house ELISA and VN assay cannot distinguish the antibody response for either EHV-1 or EHV-4. Thus, as shown in Table 3, the antibody response in the VN assay and in-house ELISA recognize EHV-1 and EHV-4 together without differentiation. The specificity and sensitivity were determined based on this situation, with the VN assay serving as the gold standard for calculation. Thus, the specificity of the in-house ELISA was determined to be 85% and the sensitivity was determined to be 100%. The similarity rate compared with the commercial ELISA was determined to be 99%. Additionally, as given in Supplementary Materials, no correlation was detected between the SN_50_ tests and the OD values of the ELISAs. Although a sera with a titer of 1/3 had an OD value of 2.158, a sera sample with a titer of 1/32 had an OD value of 0.235.

In the commercial ELISA, 74.19% of the samples tested were detected as EHV-1 positive and 86.44% as EHV-4 positive (Table 3). However, some of these positive samples were found to be negative in the VN assay for both viruses. This discrepancy may be due to the higher sensitivity of the ELISA compared to the VN assay. Pavulraj et al. (2021) stated that this could be attributed to a delayed neutralizing antibody response [17]. The relatively lower specificity (85%) of our in-house ELISA compared to the VN assay should also be considered in this context. Despite this, there is a high similarity rate of 99% between the commercial ELISA results and in-house ELISA, considering that in-house ELISA can diagnose both EHV-1 and EHV-4.

The high positivity rate in both the in-house and commercial ELISA are attributed to the endemic circulation of EHV-4 and vaccination against both viruses. The high positivity rates for the EHV-1 antigen-based in-house ELISA were due to serological cross-reactivity between EHV-1 and EHV-4. Based on all these reasons, considering the rapid growth of EHV-1 and greater stability compared to EHV-4, as well as the high compatibility of EHV-1 with commercial ELISAs, the results show 80.64% positivity for EHV-1 ELISA (125/155) and 79.35% (123/155) for EHV-4 ELISA. Consequently, the use of EHV-1 alone as an antigen in the in-house ELISA for the serological diagnosis of both viruses stands out (Table 3).

Herpesviruses cause latent infection, meaning that animals exposed to the virus retain the agent in different latency sites throughout their lives. During latent infection, the agent is reactivated in any stress situation (such as corticosteroid use, pregnancy, transportation, etc.), stimulating the immune system and making latently infected animals detectable serologically in a herd. In this way, it is possible to serologically diagnose herpesviruses, which are common and cause persistent infections. However, the most important factor that limits serological diagnosis is the inability to differentiate vaccinated and infected animals serologically due to the common vaccination. However, it is thought that the in-house ELISA developed for serological diagnosis to determine the presence of antibodies in vaccinated animals and to determine the seroprevalence of infection in unvaccinated animals can be widely used in the equine industry.

In this study, sera samples were collected from vaccinated (*n* = 98) and unvaccinated (*n* = 35) horses, and were evaluated using in-house ELISA, VN assay, and commercial ELISA (Table 4). According to these results, 98.97% of the vaccinated horse sera samples tested positive for both EHV-1 and EHV-4 using the in-house ELISA. In the VN results of the vaccinated animal samples, 71.42% (70/98) of the sera samples were positive for EHV-1, and 90.81% (89/98) were positive for EHV-4. In the commercial ELISA results of the sera samples, 88.77% of the sera samples (87/98) were positive for EHV-1, and 100% (98/98) were positive for EHV-4. According to these results, even though the ELISA and VN assay results were quite different, the in-house and commercial ELISA results were consistent, considering that the immune response against EHV-1/4 was recognized together with in-house ELISA.

Although EHV-1/4 seroprevalence is found to be quite high in both ELISA and VN assays (Table 3), the vaccinated animals SN_50_ values are found to be 1/8 for both viruses, which is quite low. This probably indicates that inactivated vaccines are not efficient in preventing the disease. Live attenuated vaccines are also not effective and are not used in Türkiye due to the potential risk of abortion in pregnant mares and the possibility of reverse virulence. Therefore, we recommend developing new vaccines using recombinant technology.

Among 35 naturally infected animals, the VN assay detected 65.71% (23/35) as positive for EHV-1 and 88.57% (31/35) as positive for EHV-4. In the in-house ELISA, 97.14% (34/35) of the sera samples were positive for EHV-1, and 94.28% (33/35) were positive for EHV-4. For the commercial ELISA, 80% (28/35) of the samples were positive for EHV-1 and 100% (35/35) were positive for EHV-4. These results indicate that most of the naturally infected animals were infected with EHV-4. The EHV-1 antigen-based in-house ELISA detected more positive cases compared to EHV-4 in-house ELISA due to serological cross-reaction. In addition, the VN assay could not recognize all of the positive cases for EHV-1/4. When evaluating the results of vaccinated and naturally infected animal samples, it was revealed that the EHV-1 in-house ELISA and commercial ELISA produced similar results in detecting antibodies against EHV-1 and EHV-4 in vaccinated and naturally infected animals.

In this study, an in-house ELISA was designed to facilitate the indirect diagnosis of EHV-1 and EHV-4, with its applicability thoroughly evaluated. The findings indicate that this ELISA provides a reliable and rapid diagnostic tool for detecting EHV-1/4, addressing some of the limitations associated with the VN assay. Although EHV-4 is more commonly observed in the field, it is recommended to use EHV-1 as the antigen in future ELISA designs, as it is easier and faster to propagate in various cell lines, benefiting from the strong cross-reactivity between EHV-1 and EHV-4.

## 5. Conclusions

In this study, an in-house ELISA was developed for the indirect diagnosis of EHV-1 and/or EHV-4, and the usability of this ELISA technique was discussed. It has been demonstrated that the in-house ELISA developed for EHV-1/4, which circulates endemically in horse populations and is controlled by vaccination in Türkiye, can be used in the rapid diagnosis of EHV-1/4 and also prevents the negatives related to VN assay. Although EHV-4 infection is more common in the field, it is suggested that EHV-1 can be used alone as an antigen in ELISA to be developed for the indirect diagnosis of EHV-1/4, because EHV-1 can be grown faster and easier in many different cell cultures, taking advantage of the strong cross-reaction between EHV-1 and EHV-4.

In conclusion, the in-house ELISA developed in this study has the potential to detect antibody responses against both EHV-1 and EHV-4 together. In addition to being able to detect antibodies against EHV-1/4, this ELISA prevents the drawbacks encountered in VNT (such as the toxic effect in sera, the need for cells, the long duration of evaluation, the need for live virus, etc.). The compatibility of the results with commercial ELISA proves the in-house ELISA’s availability.

## Figures and Tables

**Table 1:** Comparison of EHV-1 in-house ELISA results with VNT and commercial ELISA.

	EHV-1 VNT		EHV-1 Commercial ELISA		
**EHV-1 in-house ELISA**	Positive	Negative	Positive	Negative	Suspected
**Positive**	93	37	112	0	18
**Negative**	0	25	1	23	1
**Total**	93 (60%)	62 (40%)	113 (72.90%)	23 (14.83%)	19 (12.25%)
	155		155		

**Table 2:** Comparison of EHV-4 in-house ELISA results with VNT and commercial ELISA.

	EHV-4 VNT		EHV-4 Commercial ELISA		
**EHV-4 in-house ELISA**	Positive	Negative	Positive	Negative	Suspected
**Positive**	117	11	127	0	0
**Negative**	3	24	6	22	0
**Total**	120 (77.41%)	35 (22.59%)	133 (85.80%)	22 (14.20%)	0
	155		155		

**Table 3:** Comparison of EHV-1/4 in-house ELISA results with VNT and commercial ELISA.

	EHV-1/4 VNT		EHV-1/4 Commercial ELISA		
**EHV-1/4 in-house ELISA**	Positive	Negative	Positive	Negative	Suspected
**Positive**	126	5	132	0	0
**Negative**	0	24	0	23	0
**Total**	126 (81.29%)	29 (18.71%)	132 (85.16%)	23 (14.84%)	0
	155		155		

**Table 4:** Comparative evaluation of vaccinated and naturally infected animals with VNT, in-house ELISA, and commercial ELISA.

Sampled Animals	VNT EHV-1	VNT EHV-4	In-House ELISA EHV-1	In-House ELISA EHV-4	Commercial ELISA EHV-1	Commercial ELISA EHV-4	The Number of Tested Samples
Naturally infected	23 (65.71%)	31 (88.57%)	34 (97.14%)	33 (94.28%)	28 (80%)	35 (100%)	35
Vaccinated	70 (71.42%)	89 (90.81%)	97 (98.97%)	97 (98.97%)	87 (88.77%)	98 (100%)	98
Precolostral	0 (0%)	0 (0%)	0 (0%)	0 (0%)	0 (0%)	0 (0%)	16
Total	93	120	131	130	115	133	149

## Data Availability

The authors confirm that the data supporting the findings of this study are available within the article [and/or] its Supplementary Materials.

## Supplementary Materials

The following supporting information can be downloaded at: https://www.mdpi.com/article/10.3390/ani15172523/s1, Table S1: Overall ELISA and VNT results of the study.

## Abbreviations

The following abbreviations are used in this manuscript:

°C	Degrees Celsius
BSA	Bovine Serum Albumin
C.V.	Coefficient of Variation
CF	Complement Fixation
CFT	Complement Fixation Test
CO_2_	Carbon Dioxide
ED	Equine Dermis (cell line)
DLS	Dynamic Light Scattering
EHM	Equine Herpesvirus Myeloencephalopathy
EHV-1	Equine Herpesvirus Type 1
EHV-4	Equine Herpesvirus Type 4
ELISA	Enzyme-Linked Immunosorbent Assay
FCS	Fetal Calf Serum
gG	Glycoprotein G
ICTV	International Committee on Taxonomy of Viruses
IgG	Immunoglobulin G
kDa	Kilodalton
mL	Milliliter
NaCl	Sodium Chloride
OD	Optical Density
PBS	Phosphate-Buffered Saline
PDI	Polydispersity Index
PEG	Polyethylene Glycol
pH	Potential of Hydrogen
rpm	Revolutions Per Minute
SDS-PAGE	Sodium Dodecyl Sulfate–Polyacrylamide Gel Electrophoresis
SN_50_	Serum Neutralization 50% Endpoint
TCID_50_	Tissue Culture Infective Dose 50%
µg	Microgram
VN	Virus Neutralization
VNT	Virus Neutralization Test
WOAH	World Organisation for Animal Health (formerly OIE)

## References

[1] World Organisation for Animal Health (WOAH) (2017). Chapter 2.5.9.—Equine Rhinopneumonitis (Infection with Equid Herpesvirus-1 and -4). OIE Terrestrial Manual.

[2] International Committee on Taxonomy of Viruses (ICTV) Virus Taxonomy: 2022 Release. https://ictv.global/taxonomy.

[3] Davison A.J., Eberle R., Ehlers B., Hayward G.S., McGeoch D.J., Minson A.C., Pellett P.E., Roizman B., Studdert M.J., Thiry E. (2009). The Order Herpesvirales. Arch. Virol..

[4] Fukushi H., Tomita T., Taniguchi A., Ochiai Y., Kirisawa R., Matsumura T., Yanai T., Masegi T., Yamaguchi T., Hirai K. (1997). Gazelle Herpesvirus 1: A New Neurotropic Herpesvirus Immunologically Related to Equine Herpesvirus 1. Virology.

[5] Patel J.R., Heldens J. (2005). Equine Herpesviruses 1 (EHV-1) and 4 (EHV-4)–Epidemiology, Disease and Immunoprophylaxis: A Brief Review. Vet. J..

[6] Laval K., Poelaert K.C.K., Van Cleemput J., Zhao J., Vandekerckhove A.P., Gryspeerdt A.C., Garré B., van der Meulen K., Baghi H.B., Dubale H.N. (2021). The Pathogenesis and Immune Evasive Mechanisms of Equine Herpesvirus Type 1. Front. Microbiol..

[7] Slater J. (2013). Equine Herpesviruses 14. Equine Infectious Diseases E-Book.

[8] Edington N., Bridges C.G., Patel J.R. (1986). Endothelial Cell Infection and Thrombosis in Paralysis Caused by Equid Herpesvirus-1: Equine Stroke. Arch. Virol..

[9] Kydd J.H., Smith K.C., Hannant D., Livesay G.J., Mumford J.A. (1994). Distribution of Equid Herpesvirus-1 (EHV-1) in Respiratory Tract Associated Lymphoid Tissue: Implications for Cellular Immunity. Equine Vet. J..

[10] Patel J.R., Edington N., Mumford J.A. (1982). Variation in Cellular Tropism between Isolates of Equine Herpesvirus-1 in Foals. Arch. Virol..

[11] Campbell T.M., Studdert M.J. (1982). Equine Herpesvirus Type 1 (EHV1). https://www.cabidigitallibrary.org/doi/pdf/10.5555/19832218924.

[12] Slater J.D., Borchers K., Thackray A.M., Field H.J. (1994). The Trigeminal Ganglion Is a Location for Equine Herpesvirus 1 Latency and Reactivation in the Horse. J. Gen. Virol..

[13] Welch H.M., Bridges C.G., Lyon A.M., Griffiths L., Edington N. (1992). Latent Equid Herpesviruses 1 and 4: Detection and Distinction Using the Polymerase Chain Reaction and Co-Cultivation from Lymphoid Tissues. J. Gen. Virol..

[14] Paillot R., Case R., Ross J., Newton R., Nugent J. (2008). Equine Herpes Virus-1: Virus, Immunity and Vaccines. Open Vet. Sci. J..

[15] Oladunni F.S., Horohov D.W., Chambers T.M. (2019). EHV-1: A Constant Threat to the Horse Industry. Front. Microbiol..

[16] Crabb B.S., Allen G.P., Studdert M.J. (1991). Characterization of the Major Glycoproteins of Equine Herpesviruses 4 and 1 and Asinine Herpesvirus 3 Using Monoclonal Antibodies. J. Gen. Virol..

[17] Pavulraj S., Eschke K., Theisen J., Westhoff S., Reimers G., Andreotti S., Osterrieder N., Azab W. (2021). Equine Herpesvirus Type 4 (EHV-4) Outbreak in Germany: Virological, Serological, and Molecular Investigations. Pathogens.

[18] Animal Health Diagnostic Center C.U. (2021). Equine Herpesvirus-1 (EHV-1) Serum Neutralization. https://www.vet.cornell.edu/animal-health-diagnostic-center/testing-laboratories/virology/test-data/equine-herpesvirus-1-ehv-1-serum-neutralization.

[19] Telford E.A.R., Watson M.S., McBride K., Davison A.J. (1992). The DNA Sequence of Equine Herpesvirus-1. Virology.

[20] Telford E.A., Watson M.S., Perry J., Cullinane A.A., Davison A.J. (1998). The DNA Sequence of Equine Herpesvirus-4. J. Gen. Virol..

[21] Crabb B.S., MacPherson C.M., Reubel G.H., Browning G.F., Studdert M.J., Drummer H.E. (1995). A Type-Specific Serological Test to Distinguish Antibodies to Equine Herpesviruses 4 and 1. Arch. Virol..

[22] Frey H., Liess B. (1971). Vermehrungskinetik Und Verwendbarkeit Eines Stark Zytopathogenen VD-MD-Virusstammes Für Diagnostische Untersuchungen Mit Der Mikrotiter-Methode. Zentralblatt Für Veterinärmedizin Reihe B.

[23] Ludwig A.-K., De Miroschedji K., Doeppner T.R., Börger V., Ruesing J., Rebmann V., Durst S., Jansen S., Bremer M., Behrmann E. (2018). Precipitation with Polyethylene Glycol Followed by Washing and Pelleting by Ultracentrifugation Enriches Extracellular Vesicles from Tissue Culture Supernatants in Small and Large Scales. J. Extracell. Vesicles.

[24] Polson A., Keen A., Sinclair-Smith C., Furminger I.G.S. (1972). Polyethylene Glycol Purification of Influenza Virus with Respect to Aggregation and Antigenicity. Epidemiol. Infect..

[25] Möller L., Schünadel L., Nitsche A., Schwebke I., Hanisch M., Laue M. (2015). Evaluation of Virus Inactivation by Formaldehyde to Enhance Biosafety of Diagnostic Electron Microscopy. Viruses.

[26] Laemmli U.K. (1970). Cleavage of Structural Proteins during the Assembly of the Head of Bacteriophage T4. Nature.

[27] Bradford M.M. (1976). A Rapid and Sensitive Method for the Quantitation of Microgram Quantities of Protein Utilizing the Principle of Protein-Dye Binding. Anal. Biochem..

[28] Crowther J.R. (2008). The ELISA Guidebook.

[29] Xiao Y., Isaacs S.N. (2012). Enzyme-Linked Immunosorbent Assay (ELISA) and Blocking with Bovine Serum Albumin (BSA)—Not All BSAs Are Alike. J. Immunol. Methods.

[30] World Organisation for Animal Health (WOAH) (2023). Chapter 1.1.6.—Principles and Methods of Validation of Diagnostic Assays for Infectious Diseases. WOAH Terrestrial Manual 2023.

[31] Reed S.M., Toribio R.E. (2004). Equine Herpesvirus 1 and 4. Veterinary Clinics: Equine Practice.

[32] Olofsson S. (1975). Isoelectric Focusing of Herpes Simplex Virus. Arch. Virol..

[33] Konishi E., Kitai Y., Nishimura K., Harada S. (2010). Antibodies to Bovine Serum Albumin in Human Sera: Problems and Solutions with Casein-Based ELISA in the Detection of Natural Japanese Encephalitis Virus Infections. Jpn. J. Infect. Dis..

[34] El Brini Z., Fassi Fihri O., Paillot R., Lotfi C., Amraoui F., El Ouadi H., Dehhaoui M., Colitti B., Alyakine H., Piro M. (2021). Seroprevalence of Equine Herpesvirus 1 (EHV-1) and Equine Herpesvirus 4 (EHV-4) in the Northern Moroccan Horse Populations. Animals.

[35] Carvalho R., Passos L.M.F., Gouvea A.M.G., Resende M., Martins A.S., Franco G.C. (2000). Use of an ELISA System for Detection of Equine Herpesvirus 1 (EHV-1) Antibodies in Non-Symptomatic Pregnant Mares and Neonatal Foals. Arq. Bras. Med. Vet. Zootec..

[36] Afify A.F., Salem S.A.H., El-Sanousi A.A., Shalaby M. (2017). Development of a Novel Homemade ELISA Kit for Antigen Detection of EHV Causing Abortion in Egypt. https://www.researchgate.net/publication/344754905_Development_of_a_Novel_Homemade_ELISA_kit_for_Antigen_Detection_of_EHV_Causing_Abortion_in_Egypt.

