# Supplementary material for: Development of an In-House ELISA for Serological Detection of Equine Herpesvirus-1/4 Antibodies in Turkish Horses
Source: Animals (Basel). 2025 Aug 27;15(17):2523. doi: 10.3390/ani15172523 (PMC12427572; doi:10.3390/ani15172523)
Supplement: Supplementary file 1 [file animals-15-02523-s001.zip › animals-3834066-supplementary.pdf]

Table S1. Overall ELISA and VNT results of the study.

Sample no	Sample Name	EHV-1 VNT	EHV-4 VNT	In-house ELISA EHV-1	In-house ELISA EHV-4	Commercial ELISA EHV-1	Commercial ELISA EHV-4
1)	2B	Negative	Negative	0,145 (-)	0,143 (-)	0,085(-)	0,090 (-)
2)	5B	Negative	Negative	0,153 (-)	0,151 (-)	0,090 (-)	0,090 (-)
3)	3K	Negative	Negative	0,119 (-)	0,102 (-)	0,090 (-)	0,090 (-)
4)	5K	Negative	Negative	0,161 (-)	0,107 (-)	0,090 (-)	0,090 (-)
5)	6K	Negative	Negative	0,111 (-)	0,120 (-)	0,090 (-)	0,090 (-)
6)	8K	Negative	Negative	0,191 (-)	0,189 (-)	0,090 (-)	0,090 (-)
7)	18K	1/16	1/16	2,144 (+)	1,736 (+)	1,392 (+)	2,197 (+)
8)	19K	1/8	1/24	0,891 (+)	0,421 (+)	2,387 (+)	2,509 (+)
9)	20K	1/8	1/12	0,576 (+)	0,453 (+)	2,735 (+)	2,821 (+)
10)	21K	1/12	1/24	0,561 (+)	0,599 (+)	2,393 (+)	2,689 (+)
11)	22K	1/8	1/24	0,499 (+)	0,556 (+)	2,196 (+)	2,266 (+)
12)	23K	1/8	1/16	0,971 (+)	0,862 (+)	2,688 (+)	2,852 (+)
13)	1I	1/8	1/6	1,881 (+)	0,237 (+)	0,737 (+)	1,276 (+)
14)	2I	Negative	1/8	0,366 (+)	0,367 (+)	0,678 (+)	2,071 (+)
15)	4I	Negative	1/4	1,062 (+)	0,789 (+)	0,150 (?)	1,622 (+)
16)	5I	1/4	1/2	0,992 (+)	0,188 (+)	0,198 (?)	1,994 (+)
17)	6I	1/24	1/8	1,557 (+)	0,510 (+)	0,257 (+)	2,191 (+)
18)	7I	1/24	1/16	0,950 (+)	0,195 (+)	0,736 (+)	1,007 (+)
19)	8I	1/16	1/16	0,450 (+)	0,164 (-)	0,830 (+)	1,515 (+)
20)	9I	1/12	1/24	0,817 (+)	0,219 (+)	0,153 (?)	1,137 (+)
21)	10I	1/12	1/6	1,676 (+)	0,354 (+)	0,141 (?)	1,923 (+)
22)	11I	Negative	1/32	0,358 (+)	0,235 (+)	0,222 (+)	1,341 (+)
23)	12I	Negative	1/16	0,842 (+)	0,423 (+)	0,343 (+)	1,484 (+)
24)	13I	Negative	Negative	0,065 (-)	0,060(-)	0,090 (-)	0,090 (-)
25)	14I	1/12	1/4	1,203 (+)	0,233 (+)	0,643 (+)	1,737(+)
26)	15I	1/6	1/6	0,220 (+)	0,205 (+)	0,332 (+)	0,495 (+)
27)	16I	1/16	1/3	1,835 (+)	0,995 (+)	0,196 (?)	2,158 (+)
28)	17I	1/6	1/8	1,371(+)	0,206 (+)	0,224 (+)	1,558 (+)
29)	18I	1/8	Negative	1,478 (+)	1,254(+)	1,544 (+)	1,861 (+)
30)	19I	1/12	1/12	0,717 (+)	0,130 (-)	0,135 (?)	2,278 (+)
31)	20I	1/12	1/8	1,100 (+)	0,259 (+)	0,283 (+)	2,392 (+)
32)	21I	1/3	1/8	2,163 (+)	1,156 (+)	0,319 (+)	2,040 (+)
33)	22I	1/8	1/4	1,364 (+)	0,277 (+)	0,439 (+)	2,268 (+)
34)	1S	1/16	1/16	2,051 (+)	0,551 (+)	0,495 (+)	2,268 (+)
35)	2S	1/8	1/12	0,763 (+)	0,384 (+)	0,457 (+)	1,704 (+)
36)	3S	1/6	1/6	1,110(+)	0,374 (+)	0,455 (+)	2,207 (+)
37)	4S	1/3	1/8	1,161 (+)	0,463 (+)	0,236 (+)	2,109 (+)
38)	5S	1/8	1/6	1,059 (+)	0,313 (+)	1,203 (+)	2,459 (+)
39)	6S	1/4	1/4	1,077 (+)	0,240 (+)	0,532 (+)	1,934 (+)
40)	7S	Negative	1/3	1,153 (+)	0,329 (+)	0,521 (+)	1,916 (+)
41)	8S	1/4	1/6	1,541 (+)	0,341 (+)	0,351 (+)	2,168 (+)
42)	9S	Negative	Negative	0,344 (+)	0,220 (+)	0,278 (+)	2,377 (+)
43)	10S	1/16	1/12	2,080 (+)	1,266 (+)	3,237 (+)	3,477 (+)
44)	11S	1/8	Negative	0,473(+)	0,175 (+)	0,444 (+)	1,822 (+)
45)	12S	1/4	1/3	1,541(+)	0,313 (+)	0,222 (+)	2,501 (+)

46)	13S	1/12	1/8	1,475 (+)	0,305 (+)	0,270 (+)	1,973 (+)
47)	14S	1/4	1/4	1,563 (+)	0,317 (+)	0,251 (+)	1,979 (+)
48)	15S	1/12	1/6	1,325 (+)	0,236 (+)	0,730 (+)	2,304 (+)
49)	16S	1/4	1/4	2,048 (+)	0,321 (+)	0,392 (+)	2,426 (+)
50)	17S	1/6	1/6	2,061 (+)	0,606 (+)	2,982 (+)	3,215 (+)
51)	18S	1/8	1/6	0,415 (+)	0,132 (-)	0,178 (?)	2,079 (+)
52)	19S	Negative	1/4	0,448 (+)	0,304 (+)	0,172 (?)	1,268 (+)
53)	20S	1/12	1/4	0,815 (+)	0,286 (+)	0,275 (+)	1,421 (+)
54)	21S	1/6	Negative	1,944 (+)	0,393 (+)	0,315 (+)	2,145 (+)
55)	22S	1/24	Negative	1,026 (+)	0,230 (+)	0,219 (+)	2,189 (+)
56)	23S	1/6	1/6	2,234 (+)	0,624 (+)	0,264 (+)	2,135 (+)
57)	30S	1/4	1/12	2,341 (+)	1,424 (+)	0,169 (?)	1,622 (+)
58)	31S	1/6	1/6	0,794 (+)	1,301 (+)	1,704 (+)	2,737 (+)
59)	32S	1/6	1/6	0,557 (+)	1,084 (+)	2,902 (+)	2,920 (+)
60)	33S	1/8	1/12	1,071 (+)	2,000 (+)	3,152 (+)	3,334 (+)
61)	34S	Negative	1/8	0,678 (+)	0,531 (+)	0,167 (?)	1,952 (+)
62)	35S	1/8	Negative	2,354 (+)	1,444 (+)	3,166 (+)	3,587 (+)
63)	36S	1/4	1/6	0,622 (+)	1,341 (+)	1,311 (+)	1,411 (+)
64)	37S	Negative	Negative	0,128(-)	0,100 (-)	0,176 (?)	0,181 (-)
65)	38S	1/8	1/3	1,404 (+)	0,877 (+)	0,658 (+)	2,481 (+)
66)	39S	Negative	1/6	0,913 (+)	1,558 (+)	3,132 (+)	3,340 (+)
67)	40S	Negative	1/3	1,045 (+)	1,738 (+)	3,299 (+)	3,404 (+)
68)	41S	Negative	1/6	1,037 (+)	0,711 (+)	0,177 (?)	1,386 (+)
69)	48S	1/16	1/16	2,465 (+)	1,976 (+)	0,322 (+)	0,856 (+)
70)	49S	Negative	1/24	0,552(+)	1,073 (+)	2,992 (+)	2,998 (+)
71)	50S	1/8	Negative	2,365 (+)	1,826 (+)	0,551 (+)	2,414 (+)
72)	Merida	Negative	1/6	0,344 (+)	0,266 (+)	0,861 (+)	1,111 (+)
73)	Özdem	Negative	Negative	0,390 (+)	0,216 (+)	0,389 (+)	1,729 (+)
74)	Kesikkulak	Negative	1/8	0,765 (+)	0,271 (+)	0,448 (+)	1,173 (+)
75)	Karatay	Negative	1/6	0,766 (+)	0,541 (+)	0,665 (+)	1,327 (+)
76)	Ponçik	Negative	Negative	0,163 (-)	0,168 (-)	1,017 (+)	1,714 (+)
77)	Karayel	Negative	Negative	0,327 (+)	0,206 (+)	0,251 (+)	2,229 (+)
78)	Kuki	Negative	1/12	1,563(+)	1,122 (+)	0,174 (?)	1,786 (+)
79)	İnci	Negative	1/4	0,501 (+)	0,238 (+)	0,396 (+)	1,989 (+)
80)	1TG	Negative	1/8	0,721 (+)	0,350 (+)	0,136 (?)	0,729 (+)
81)	2TG	Negative	1/4	0,403 (+)	0,269 (+)	0,129 (?)	0,228 (+)
82)	3TG	Negative	1/8	1,114 (+)	1,708 (+)	0,136 (?)	0,633 (+)
83)	4TG	Negative	1/12	0,356 (+)	0,397 (+)	0,218 (+)	0,945 (+)
84)	5TG	Negative	1/12	0,536 (+)	0,230 (+)	0,204 (+)	0,529 (+)
85)	6TG	Negative	1/8	0,917 (+)	0,650 (+)	0,313 (+)	0,887 (+)
86)	7TG	Negative	1/6	1,158 (+)	0,789 (+)	0,119 (?)	1,014 (+)
87)	8TG	Negative	1/12	1,236 (+)	0,931 (+)	0,434 (+)	0,974 (+)
88)	9TG	Negative	1/12	0,864 (+)	0,652 (+)	0,193 (?)	0,442 (+)
89)	10TG	Negative	1/12	1,559 (+)	1,324 (+)	0,162 (?)	0,762(+)
90)	11TG	1/12	1/16	2,555 (+)	1,584 (+)	0,495 (+)	2,133 (+)
91)	12TG	1/16	1/24	2,155 (+)	1,662 (+)	0,447 (+)	1,651(+)
92)	13TG	1/24	1/24	2,173 (+)	1,569 (+)	0,462 (+)	2,055 (+)
93)	14TG	Negative	1/4	1,896 (+)	1,384 (+)	0,306 (+)	1,786 (+)
94)	15TG	Negative	1/24	2,084 (+)	1,331 (+)	0,666 (+)	1,014 (+)
95)	16TG	1/16	1/16	1,600 (+)	1,973 (+)	0,429 (+)	2,011 (+)

96)	17TG	1/32	1/8	2,555 (+)	1,640 (+)	1,083 (+)	1,780 (+)
97)	18TG	1/48	1/24	2,086 (+)	1,661 (+)	0,384 (+)	1,192 (+)
98)	19TG	1/16	1/12	1,390 (+)	1,455 (+)	0,380 (+)	1,464 (+)
99)	20TG	1/32	1/48	2,075 (+)	1,283 (+)	0,815 (+)	1,061 (+)
100)	21TG	1/24	1/4	1,864 (+)	1,329 (+)	0,460 (+)	1,892 (+)
101)	22TG	1/4	1/48	2,084 (+)	1,788 (+)	0,530 (+)	1,074 (+)
102)	23TG	1/32	1/12	1,201 (+)	2,336 (+)	0,280 (+)	1,482 (+)
103)	24TG	1/16	1/16	2,204 (+)	1,705 (+)	1,855 (+)	2,186 (+)
104)	25TG	1/32	1/12	1,202 (+)	1,311 (+)	0,430 (+)	1,885 (+)
105)	26TG	1/16	1/4	2,063 (+)	0,961 (+)	1,227 (+)	1,586 (+)
106)	27TG	1/12	1/4	2,059 (+)	1,684 (+)	1,328 (+)	1,721 (+)
107)	28TG	1/16	1/64	2,273 (+)	1,691 (+)	0,645 (+)	2,465 (+)
108)	29TG	1/12	1/16	2,081 (+)	1,614 (+)	1,146 (+)	1,284 (+)
109)	30TG	1/12	1/24	2,360 (+)	1,609 (+)	0,411 (+)	1,190 (+)
110)	31TG	1/16	1/6	2,132 (+)	1,375 (+)	0,233 (+)	1,941 (+)
111)	32TG	1/8	1/24	2,448 (+)	2,219 (+)	0,569 (+)	2,102 (+)
112)	33TG	1/8	1/4	2,656 (+)	1,564 (+)	1,105 (+)	1,125 (+)
113)	34TG	1/8	1/32	2,333 (+)	1,758 (+)	1,313 (+)	3,235 (+)
114)	35TG	1/6	1/12	2,334 (+)	1,816 (+)	0,291 (+)	1,883 (+)
115)	36TG	1/8	1/8	2,365 (+)	2,336 (+)	1,642 (+)	2,971 (+)
116)	37TG	1/8	1/8	2,133 (+)	1,607 (+)	1,241 (+)	3,038 (+)
117)	38TG	1/8	1/8	1,476 (+)	2,037 (+)	2,962 (+)	3,135 (+)
118)	39TG	1/12	1/12	2,164 (+)	1,624 (+)	2,180 (+)	2,283 (+)
119)	40TG	1/8	1/8	1,197 (+)	1,882 (+)	2,795 (+)	3,244 (+)
120)	41TG	1/8	1/8	1,003 (+)	1,634 (+)	2,373 (+)	3,305 (+)
121)	42TG	1/8	1/8	0,604 (+)	1,117 (+)	3,314 (+)	3,298 (+)
122)	43TG	1/8	1/8	0,537 (+)	0,846 (+)	2,592 (+)	2,739 (+)
123)	44TG	1/8	1/8	0,925 (+)	1,682 (+)	3,526 (+)	3,239 (+)
124)	45TG	1/8	1/8	1,055 (+)	1,966 (+)	3,418 (+)	3,357 (+)
125)	46TG	1/8	Negative	2,091 (+)	2,189 (+)	0,356 (+)	2,342 (+)
126)	47TG	1/48	1/48	2,308 (+)	1,321 (+)	2,500 (+)	2,505 (+)
127)	48TG	1/8	1/12	0,889 (+)	1,960 (+)	3,519 (+)	3,407 (+)
128)	49TG	Negative	Negative	0,844 (+)	0,630 (+)	0,216 (+)	1,747 (+)
129)	50TG	1/4	1/8	1,713 (+)	0,132 (-)	0,418 (+)	1,808 (+)
130)	51TG	Negative	1/16	1,551 (+)	1,548 (+)	0,227 (+)	1,231 (+)
131)	52TG	Negative	1/12	1,478 (+)	1,278 (+)	0,388 (+)	0,777 (+)
132)	53TG	1/8	1/12	2,055 (+)	1,672 (+)	0,225 (+)	1,203 (+)
133)	54TG	1/4	1/12	1,558 (+)	0,970 (+)	0,350 (+)	2,304 (+)
134)	55TG	Negative	1/16	1,527 (+)	1,118 (+)	0,310 (+)	0,545 (+)
135)	56TG	Negative	1/12	1,152 (+)	1,137 (+)	0,352 (+)	1,004 (+)
136)	57TG	1/4	1/8	2,071 (+)	1,730 (+)	1,730 (+)	1,730 (+)
137)	58TG	Negative	1/16	1,176 (+)	0,899 (+)	0,265 (+)	1,390 (+)
138)	59TG	Negative	1/12	1,856 (+)	1,798 (+)	0,327 (+)	0,862 (+)
139)	60TG	1/8	1/24	1,889 (+)	1,161 (+)	0,339 (+)	1,405 (+)
140)	1PRE	Negative	Negative	0,121 (-)	0,138 (-)	0,073 (-)	0,096 (-)
141)	2PRE	Negative	Negative	0,135(-)	0,131 (-)	0,086 (-)	0,093 (-)
142)	3PRE	Negative	Negative	0,125(-)	0,119 (-)	0,075 (-)	0,068 (-)
143)	4PRE	Negative	Negative	0,114 (-)	0,153 (-)	0,087 (-)	0,082 (-)
144)	5PRE	Negative	Negative	0,165 (-)	0,123 (-)	0,087 (-)	0,083(-)
145)	6PRE	Negative	Negative	1,393 (+)	1,433 (+)	0,343 (+)	1.900 (+)

146)	7PRE	Negative	Negative	0,155 (-)	0,130 (-)	0,083 (-)	0,084 (-)
147)	8PRE	Negative	Negative	0,143 (-)	0,143 (-)	0,078 (-)	0,087 (-)
148)	9PRE	Negative	Negative	0,156 (-)	0,126 (-)	0,065 (-)	0,066 (-)
149)	10PRE	Negative	Negative	0,156 (-)	0,132 (-)	0,073 (-)	0,093 (-)
150)	11PRE	Negative	Negative	0,152 (-)	0,123 (-)	0,065(-)	0,073 (-)
151)	12PRE	Negative	Negative	0,151 (-)	0,133 (-)	0,093 (-)	0,096 (-)
152)	13PRE	Negative	Negative	0,135 (-)	0,120 (-)	0,073 (-)	0,063 (-)
153)	14PRE	Negative	Negative	0,125 (-)	0,132 (-)	0,088 (-)	0,087 (-)
154)	15PRE	Negative	Negative	0,147 (-)	0,137 (-)	0,079 (-)	0,084 (-)
155)	16PRE	Negative	Negative	0,158 (-)	0,124 (-)	0,074 (-)	0,065 (-)
